# Genome Sequencing to Prevent Hospital-Acquired Infections Caused by Carbapenem-Resistant Acinetobacter baumannii Due to Importation and Intra-facility Transmission in a Regional Hospital Network: Study Protocol for Implementation Research

**DOI:** 10.7759/cureus.94812

**Published:** 2025-10-17

**Authors:** Basanti Pathi, Jyoti Prakash Sahoo, Ashoka Mahapatra, Rajashree Panigrahy, Sanghamitra Padhi, Priya Ranjan Lenka, Indrani Mohanty, Balamurugan Ramadass, Kumudini Panigrahi, Shikha Dixit, Manoja K Das

**Affiliations:** 1 Microbiology, Kalinga Institute of Medical Sciences, Bhubaneswar, IND; 2 Pharmacology, Kalinga Institute of Medical Sciences, Bhubaneswar, IND; 3 Microbiology, All India Institute of Medical Sciences, Bhubaneswar, Bhubaneswar, IND; 4 Microbiology, Institute of Medical Sciences (IMS) Siksha 'O' Anusandhan University Medical Hospital, Bhubaneswar, IND; 5 Microbiology, Maharaja Krushna Chandra Gajapati Medical College and Hospital, Brahmapur, IND; 6 Microbiology, Saheed Laxman Nayak Medical College and Hospital, Koraput, IND; 7 Microbiology, Pandit Raghunath Murmu Medical College and Hospital, Baripada, IND; 8 Biochemistry, All India Institute of Medical Sciences, Bhubaneswar, Bhubaneswar, IND; 9 Epidemiology, The INCLEN Trust International, New Delhi, IND; 10 Public Health, The INCLEN Trust International, New Delhi, IND

**Keywords:** antibiotic susceptibility test, antimicrobial resistance, carbapenem-resistance, hospital-acquired infection, implementation research, inter-facility transmission, metagenomics, multilocus sequence typing (mlst), plan-do-study-act, acinetobacter baumannii

## Abstract

Background and objectives: Carbapenem-resistant *Acinetobacter baumannii* (CRAb) is among India's leading bacteria responsible for hospital-acquired infections (HAIs). The CRAb causing intra-facility and inter-facility (or importation) HAI transmissions may differ phylogenetically. For control and prevention of CRAb-associated HAIs within and across facilities, information about the phylogenetic lineage characterization and contextual risk factors is critical. To our knowledge, there is no preliminary state-level data available from Odisha state in India regarding the dynamics of CRAb transmission (intra- and inter-facility), phylogenetic lineages, risk factors, and geospatial epidemiology. This study shall document the dynamics of CRAb-associated HAIs, the phylogenetic lineages responsible for intra-facility and inter-facility transmissions, and the risk factors. We shall leverage the CRAb phylogenetic data and risk factors identified through an integrated laboratory-clinical-epidemiological-genomic surveillance for tackling the intra-facility and inter-facility transmissions and outbreaks using implementation research approaches with adoption of contextualized hospital infection control and prevention measures and antibiotic stewardship efforts in a hospital network in Odisha state, India.

Methods: This study adopts an integrated prospective facility-based surveillance with a quasi-experimental design using the plan-do-study-act (PDSA) implementation research cycles with mixed-methods data collection approaches. The study will be conducted over three years at six tertiary care medical institutions in Odisha. Prospective surveillance over 24 months at these hospitals will monitor the CRAb isolates to identify HAI outbreaks and intra- and inter-facility transmissions. The metagenomic and genome-wide sequencing (GWS) shall document CRAb phylogenetic lineages for outbreaks and intra- and inter-facility transmissions. Geospatial mapping shall document the spatial characterization of CRAb transmission for the index cases. Formative research shall identify potential risk factors at various levels for HAIs, outbreaks, intra- and inter-facility CRAb transmissions, hospital infection prevention, surveillance, and antibiotic stewardship efforts, using the Consolidated Framework for Implementation Research (CFIR). Based on this information, contextualized strategies and interventions to strengthen hospital infection prevention, surveillance, and antibiotic stewardship efforts shall be implemented at these facilities using incremental PDSA cycles. Data from these PDSA cycles shall be compared to document the impact on CRAb outbreaks and process indicators. The practices' and interventions' feasibility, acceptability, and sustainability shall be documented.

Results: We do not have any observations, as we have not started the study yet.

Conclusion: The study shall generate evidence on genoepidemiology, transmission dynamics of HAIs due to CRAb in Odisha, India, and the associated risk factors. The lessons from context-specific implementation strategies (covering infection surveillance, prevention strategies, and antibiotic stewardship domains) to tackle CRAb-associated HAIs outbreaks and the feasibility, acceptability, and sustainability shall inform the policy and practices. The geographic signatures of horizontal CRAb-associated HAIs will generate a new knowledge base to design future intervention measures.

## Introduction

Hospital-acquired infection (HAI) caused by multidrug-resistant (MDR) *Acinetobacter baumannii* (*A. baumannii*) strains is a global health concern [1]. MDR in *A. baumannii i*s attributed to the expression of virulence factors and propensity to acquire resistance genes through different mobile genetic elements (MGEs) [1,2]. Globally, carbapenem-resistant *A. baumannii *(CRAb) are classified under 'Urgent Threat' and 'Priority-1: Critical pathogens' [3]. High prevalence (40%-85%) of CRAb-associated severe HAIs is a major healthcare challenge in India [4,5].

Desiccation resistance enables longer survival of CRAb on inanimate surfaces, contributing to intra-facility transmission and outbreaks [2,5]. Additionally, the transfer or referral of patients hastens inter-facility transmission, CRAb importation, and clonal spread. Apart from the nosocomial infections, community-acquired CRAb infections are also being reported, which aggravates the concern [6-8]. Intrinsic and excessive genome plasticity-mediated acquisition of MGEs or antibiotic resistance genes (ARGs) makes treating CRAb difficult. Carbapenem resistance in *A. baumannii* is mediated by genes encoding class-D oxacillinases (blaOXA-23-like/blaOXA-51-like/blaOXA-58-like), carried on many MGEs. Additionally, horizontally transferred genes contribute significantly to the MDR phenotype of *A. baumannii *[6-9].

Inter-facility patient transfers have been linked to transmission and outbreaks of HAIs due to CRAb. The CRAb responsible for intra- and inter-facility transmissions may differ in phylogenetic lineages [5,10-12]. Routine hospital infection surveillance focuses on bacterial sources and antimicrobial susceptibility testing (AST) profiles and cannot distinguish between intra- and inter-facility (importation) transmissions. Thus, to control the nosocomial pathogens within and across healthcare facilities, adopting appropriate preventive and interventional measures and a better understanding of CRAb clonal and phylogenetic lineages are critical. High-resolution phylogenetic information will immensely help map strategies and implement targeted actions [13-15]. With the changing CRAb prevalence and emergence of outbreaks, strategies for surveillance and interventions need refinement [6-9].

Despite studies documenting the burden of HAI, no data are available from India about the dynamics of intra- or inter-facility CRAb transmission, clonal and phylogenetic lineages, and geospatial epidemiology. Also, there is no evidence on the impact of the control and prevention efforts to tackle the intra- or inter-facility transmissions of CRAb-associated HAI. Although CRAb-associated HAI is a significant public health problem in India, the lack of phylogenomic information makes tracking its persistence and transmission (intra- or inter-facility) dynamics difficult. Metagenomics and whole genome sequencing (WGS) document CRAb phylogenetic/clonal/epidemiological relatedness and prevailing resistance mechanisms [16,17]. Investigating the clonal nature of CRAb isolates will provide valuable data for infection management programs.

We propose to study the CRAb intra- and inter-facility transmission dynamics, including the clonal/phylogenetic lineages, and implement intervention strategies to tackle and prevent their outbreaks in a network of hospitals in Odisha, India. Implementing contextualized, codeveloped infection control and prevention strategies informed by integrated laboratory-clinical-epidemiological-genomic surveillance within a network of tertiary care hospitals in Odisha shall reduce the intra-facility and inter-facility transmission of CRAb.

## Materials and methods

Study sites

The study sites include six academic tertiary care hospitals (medical colleges) in Odisha, India, which follow intra-state referral patterns. The study sites are: 1. Kalinga Institute of Medical Sciences, Bhubaneswar, Odisha; 2. All India Institute of Medical Sciences, Bhubaneswar, Odisha; 3. Institute of Medical Sciences (IMS) Siksha 'O' Anusandhan University Medical Hospital, Bhubaneswar, Odisha; 4. Maharaja Krushna Chandra Gajapati Medical College, Berhampur, Odisha; 5. Saheed Laxman Nayak Medical College, Koraput, Odisha; and 6. Pandit Raghunath Murmu Medical College, Baripada, Odisha. While three hospitals are located in one city (Bhubaneswar), the other three are in three cities (Berhampur, Koraput, and Baripada) separated by 180-500 km. These hospitals have well-functioning microbiology laboratories and active hospital infection surveillance and antimicrobial stewardship programs (AMSP). Including the major tertiary care institutions in the state, a mixture of public and private facilities with geographic representation shall enable obtaining a comprehensive picture of the state regarding the CRAb intra- and inter-facility transmission dynamics.

Study design and overview

This multi-centre study adopts a blend of prospective facility-based surveillance integrated with implementation research using a quasi-experimental study design with mixed-methods data collection. The Consolidated Framework for Implementation Research (CFIR) with the iterative plan-do-study-act (PDSA) improvement cycles shall be used for the implementation research. For outcome assessment, the pre-post approach shall compare the indicators between the baseline and endline cycles and between the cycles. The study shall be done in three phases: (1) immersion and formative research to understand the status and system as they are and identify the factors and gaps; (2) co-development and implementation of site-specific contextual strategies and actions integrated with surveillance for CRAb; and (3) outcome assessment and documentation of feasibility, acceptability, and sustainability.

Study participants and stakeholders

The patients admitted to these study hospitals with infections caused by *A. baumannii* shall be the participants. Out of them, while the patients with CRAb isolates shall be the index cases, the patients with carbapenem-sensitive *A. baumannii* (CSAb) isolates shall be considered as the comparison to document the risk factors and pattern of transmission dynamics. The study stakeholders shall include doctors, nurses, support staff from different departments/units (wards-medical, pediatrics, surgical, and ICUs), the hospital infection control committee (HICC), AMSP team members, pharmacists, and hospital administrators. From some of the implementers (doctors, nurses, and support staff), data related to infection prevention and control (IPC) and AMSP practices shall be collected.

Study team

The study team consists of (a) the research team and (b) the implementation team. These teams will co-develop the site-specific contextual strategies and actions.

Research Team

This group comprises investigators and research staff who will be primarily responsible for formative research, supporting the implementation of the intervention, conducting surveillance, performing data collection for iterative PDSA cycles, and assessing the outcomes.

Implementation Team

The care providers (doctors, nurses, and support staff from different departments/units (wards-medical, pediatrics, and surgical; ICUs), the HICC and AMSP team, pharmacists, and hospital administrators form this team. The implementation team will primarily be responsible for implementing the co-developed strategies and interventions, as per their responsibilities within the hospital setting.

Immersion and formative research

This phase aims to conduct a situational analysis to understand the current practices related to IPC, antibiotic usage in the different wards/units/ICUs, HICC, and AMSP, and identify potential barriers, facilitators, and key influencers. Data collection shall include (i) HICC and AMSP practices in the hospital and specific wards/units/ICUs with and without CRAb isolates during the last six months and (ii) interaction with the key stakeholders, including care providers (doctors-faculty and residents, nurses, and support staff) from different departments (medical, surgical, and ICUs), HICC, AMSP committee members, pharmacists, and hospital administrators (30 interactions per hospital, guided by data saturation). Additionally, best practices and examples from literature and guidelines shall be collated.

Co-development, optimization, and implementation of strategies and interventions

Based on the formative research findings, the areas of concern shall be identified, and a conceptual framework will be co-developed (including the research team and implementation team) to frame the appropriate strategies and intervention actions to address them. Visual mapping of HAI, IPC, AMSP, and surveillance components and their relationships and interactions shall be done to assist the model's co-development. Co-design workshops will be held in each hospital involving the key stakeholders to co-develop the model '0' using the key domains: asepsis practices, safety culture, protocols/guidelines, antibiotic stewardship, device disinfection, environmental hygiene, hand hygiene, surveillance, patient screening and movement, leadership, and information systems. Generic and domain-specific strategies and interventions will be developed at the system level, ward/ICU/unit level, and individual level.

The core components of the provisional model shall target (a) enhancing the safety culture among healthcare providers, (b) reinforcing AMSP, HICC, and IPC activities, (c) streamlining patient triaging and movement, and (d) data-driven decision-making. The workflows for the strategies, interventions, and indicators to monitor their implementation and outcome shall be identified. The provisional model (M0) will be implemented in a limited setting (one ICU and one ward, based on the CRAb isolation trend) at each site following PDSA cycles. Quantitative data on key indicators focusing on the implementation strategy (e.g., trainings, IPC, AMSP, clinical care, surveillance), process indicators (e.g., protocol adherence, antibiotic use, disinfection practices, patient flow management), and implementation measures (e.g., acceptability, feasibility) and qualitative data (e.g., formal and informal interactions with stakeholders) will be collected.

Pre- and post-implementation data will be used to gauge acceptability, feasibility, and effectiveness based on process and quality outcome measures. Based on the findings and feedback, the model will be refined, and key areas where it has not worked effectively will be identified. Subsequent cycles with necessary model adjustments will be reiterated. We anticipate three iterative processes to refine and optimize the model (M1). Implementation of the M1 model shall be expanded to other wards/units/ICUs following continuous incremental PDSA cycles. We plan to implement five quarterly exponential PDSA cycles, coupled with documentation of the data focusing on the strategy, process, implementation, and outcome indicators; qualitative data (in-depth interviews, n=20, driven by data saturation); and the surveillance data for the CRAb outbreak and intra- and inter-facility transmissions. Based on the learning, the strategies/actions/processes will be refined. Appropriate capacity building and mentoring for the care providers and stakeholders shall be provided to implement strategies and interventions.

Implementation research conceptual frameworks

**Figure 1:**
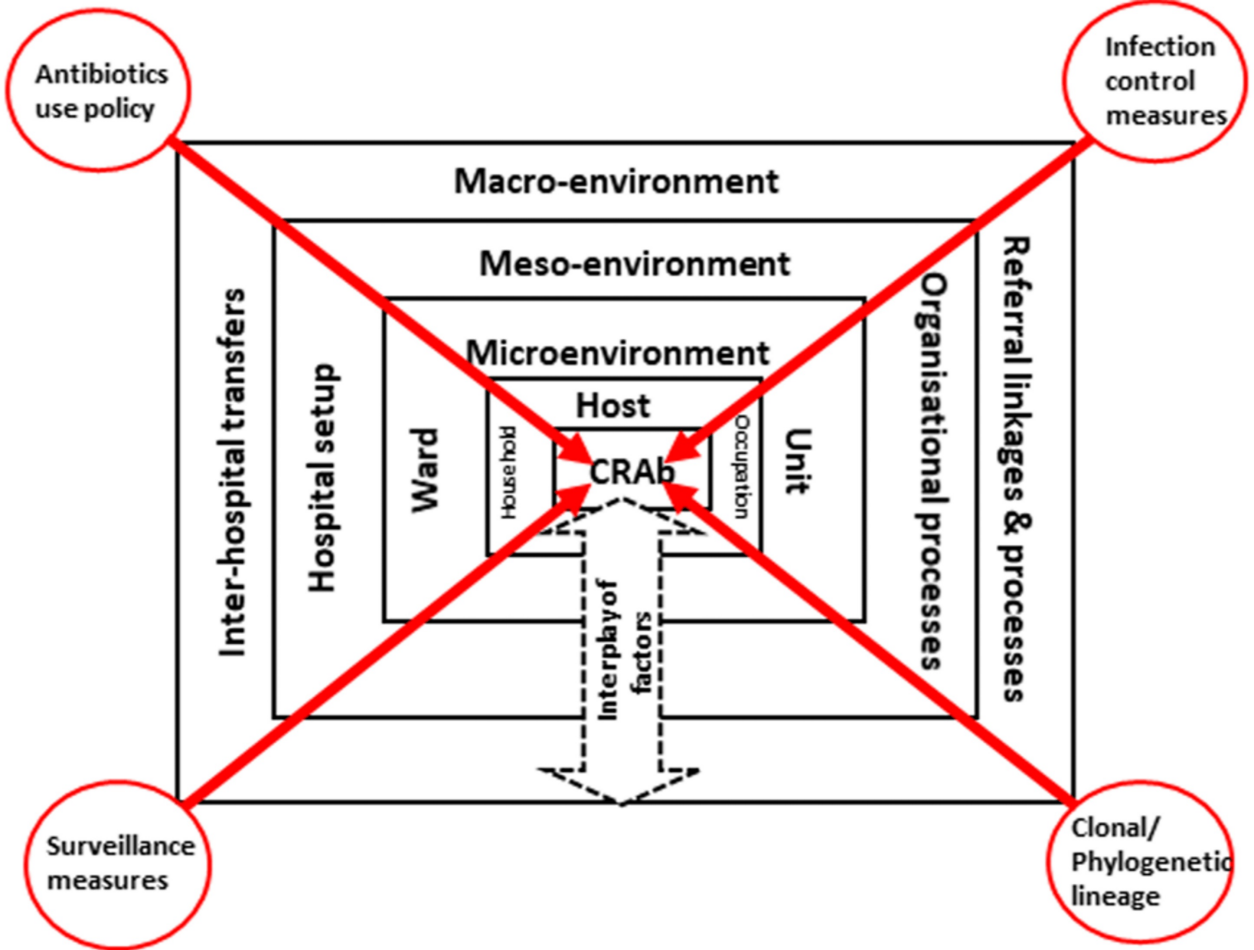
The conceptual domains of the factors influencing HAIs due to CRAb The figure has been created by Manoj K. Das. Unit and ward indicate the specific clinical units, wards, or ICUs within the same hospital. HAI: hospital-acquired infection; CRAb: carbapenem-resistant *Acinetobacter baumannii*; ICU: intensive care unit.

The CFIR and its constructs will examine the context, determinants, barriers, facilitators, feasibility, and acceptability. The determinants/barriers/facilitators shall focus on four key domains (IPC, AMSP/antibiotic use, surveillance, and CRAb transmission risks), four levels (host/patient, ward/unit/ICU, intra-hospital, and inter-hospital), and three risk-based patient flow processes (ward-level bed allocation, intra-hospital transfers/movements, and inter-hospital referrals) (Figure 1). Patient-level factors like sociodemography, occupation, and household will also be explored. The factors influencing HAI and CRAb transmission shall be organized under micro-environment (ward/ICU/unit level), meso-environment (hospital/inter-department levels, organizational processes), and macro-environment (inter-hospital transfer/referral processes) levels. Table 1 shows the framework for implementing high-quality care throughout the study period.

**Table 1:** Determinant framework used for implementing high-quality care CFIR: consolidated framework for implementation research; HAI: hospital-acquired infection; HICC: hospital infection control committee; IPC: infection prevention and control; AMSP: antimicrobial stewardship Program; ICU: intensive care unit; IT: information technology

CFIR domain	CFIR construct	The determinants and direction of influence
Intervention characteristics	-	Enable the infection control nurse's function.
		Prioritize HAI surveillance and data-driven action.
		Collect, collate, and analyze the HAI surveillance data.
		Nudge the optimal function of the HICC.
		Streamline the AMSP activities at various levels.
		Advocacy for IPC as a priority for the administrator
Inner Setting	Policy and laws	Safety culture and infection control as priorities for the hospital.
		Recognition of HICC, AMSP, and surveillance as a priority.
		Written policy and protocol for IPC, AMSP, and monitoring.
		Policy and protocols for risk-based patient triaging, movement, and transfers.
	Infrastructure	Physical infrastructure, space, and materials.
	Financing	Resource (budget, supplies, and logistics) allocation for IPC, HICC, and AMSP.
	Culture	Recognition of the role of the infection control nurse and acceptance by hospital administration.
	Work infrastructure	Availability of an adequate number of full-time infection control nurses (as per norm) and workload.
		Support from ward management, clinical nurses, and unit/ICU in-charges.
		Functional HICC, AMSP, and periodic surveillance conduct and review platform.
	IT infrastructure	Data management, archival, analysis, and reporting.
	Access to knowledge	Opportunities for training and refresher training on IPC, AMSP, and safety culture.
	Communication	Sharing of IPC, HAI, and AMSP findings with the stakeholders.
Outer Setting	Policies and laws	HAI as a priority for the state and national authority.
		Reporting requirements on HAI from hospitals.
		Guidelines on AMSP and antibiotic usage for various conditions.
		Recognition of hospitals as per the implementation of the HAI and IPC protocols.
	Quality benchmarking	Inclusion of HAI as part of the hospital quality benchmarking and accreditation.
	Financing	Budget allocation for IPC, HICC, and AMSP activities.
	Local attitudes	Antibiotic usage and care-seeking practices.
Individual characteristics	Implementation team members	Knowledge, attitude, and competency of the infection control nurse.
		Motivation of the infection control nurse.
		Attitude of clinicians and nurses towards IPC and surveillance.
		Attitude of pharmacists towards antibiotic use surveillance.
	Mid-level leaders	Attitude of ward/unit/ICU in-charge/leader for IPC, surveillance, HICC, and AMSP activities.
	High-level leaders	Attitude of hospital administrator for IPC, surveillance, HICC, and AMSP activities.
Implementation process	Reflecting	Reviews and audits of key services and function areas.
	Assessing context	Assessing context, barriers, and facilitators.
	Teaming	Joint planning, team building, and collaborating to implement specific tasks.
		Implementation of IPC, surveillance, HICC, and AMSP activities, and periodic monitoring.
		Platform for joint review of the findings and experiences.
	Access to knowledge	Conduct regular/periodic training and refresher orientation of various staff.
	Communication	Conduct of HICC and feedback to the units/wards/ICUs and hospital administration.
	Positive deviance	Recognize opinion leaders and local champions and share/disseminate best practices.

Hospital-level surveillance

Prospective integrated laboratory-clinical-epidemiological-genomic surveillance shall be conducted over 24 months at the network hospitals. For the patients with *A. baumannii* isolates, individual-level and unit/ward/ICU-level data shall be collected. Data from patients with CRAb isolates (index cases) and CSAb isolates (comparison/controls, isolates within one week from the same ward) shall be compared to identify the risk factors. The modifiable hospital-level and individual-level risk factors shall be targeted for intervention during the PDSA cycles. For the patients, data for clinical parameters (diagnosis, co-morbidities, past illnesses, medications/antibiotics use), investigations (microbiology, others), treatment (antibiotics, biologicals, procedures, ancillary care), referrals, hospital outcome, and sociodemography shall be collected. The geospatial mapping of the patient's residence (area) and hospitals attended shall be done. For the wards/units/ICUs, data on bed occupancy, patient-nurse ratio, and IPC surveillance shall be collected.

**Figure 2:**
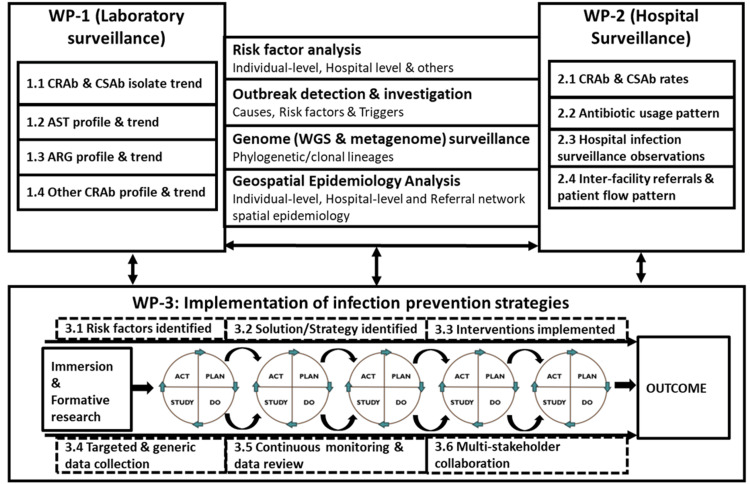
Overall project framework, components (work panels) and linkages The figure has been created by Manoj K. Das. The schematic diagram illustrates the three work panels, i.e., laboratory surveillance, hospital surveillance, and implementation of strategies. WP: work panel; CRAb: carbapenem-resistant *Acinetobacter baumannii*; CSAb: carbapenem-sensitive *Acinetobacter baumannii*; AST: antimicrobial susceptibility testing; ARG: antibiotic-resistant gene; WGS: whole genome sequencing; PDSA: plan-do-study-act

Data on hospital infection surveillance (isolates, IPC, AMPS, and antibiotic use) shall be collected periodically. Any CRAb outbreak identified shall be investigated (jointly with the HICC) to identify the potential sources/risk factors. Metagenomics and whole genome sequencing (WGS) of strategically selected CRAb isolates (for outbreaks pre-, during, and post-outbreak periods and suspected intra- and inter-hospital transmission) shall be done. Based on the metagenomics sequence data, the phylogenetic lineage and clonal lineages for the clonal complexes of CRAb and antibiotic resistance genes (ARGs) shall be mapped. The overall organization of surveillance components (two work panels (WP), WP-1: laboratory surveillance for bacteria characteristics and WP-2: hospital surveillance for patient, unit/ward/ICU, and hospital-level data) and linkages with implementation of strategies and interventions (WP-3, formative research, PDSA cycles, and outcome) are shown in Figure 2.

Evaluation and outcome documentation

The primary outcome of the implementation is to document the change in the CRAb outbreaks and associated HAIs at the network hospitals and the inter-facility transmissions. Each hospital shall be the unit for outcome documentation. Summative evaluation shall compare the indicators from baseline and endline periods/cycles (each three months) to assess the impact and implementation outcomes. The concurrent assessments shall use laboratory and hospital surveillance and implementation-specific indicators data for the quarterly PDSA cycles to monitor progress and inform implementation refinements.

Primary Outcome Indicators

The primary outcome indicators shall include (i) the number of CRAb outbreaks every quarter at the hospitals; (ii) the proportion of CRAb-associated HAIs in the wards/units/ICUs every quarter; and (iii) the proportion of CRAb-associated HAIs identified as potential inter-facility transmission.

Secondary Outcome Indicators

The secondary outcome indicators shall focus on hospital and ward/unit/ICU preparedness for HAI prevention and outbreak responses. The proposed indicators shall include (i) detection of HAIs (bloodstream infections, surgical site infections, ventilator-associated pneumonias, catheter-associated urinary tract infections); (ii) rational antibiotic use; (iii) hand hygiene practices; (iv) invasive line and catheter insertion and handling practices; (v) unit-level sanitation (surface) and disinfection (reusable devices) practices; (vi) biomedical waste management; (vii) IPC, AMSP, and HICC surveillance; and (viii) knowledge and attitude status of care providers.

Implementation Outcome Indicators

Assessment of model implementation will use the parameters of acceptability (aggregable for stakeholders), adoption (initiation by stakeholders), appropriateness (perceived fit and relevance for setting), feasibility (utilization in routine practice), penetration (used by stakeholders and units/wards/ICUs), and sustainability (maintenance of practices).

Process Outcome Indicators

These indicators will be used to monitor, optimize, and document the quality of model implementation. These will be specific to the strategies and interventions identified, and inputs for co-development and refinements.

The baseline (three months prior to implementation), concurrent evaluations (during implementation), and end-line (three months after completion of five PDSA cycles) data will be analyzed to assess the changes in the outcome indicators, feasibility, acceptability, and sustainability of the model implementation. The sampling strategy and methodology for data collection will be similar for the baseline and endline periods. With an anticipated decline in the proportion of inter-facility CRAb transmission episodes from 30% (baseline) to 15% (endline), 118 CRAb isolates among hospitalized patients shall be needed per group. The sample size for the endline may be revised based on baseline findings.

Data collection and management

The laboratory and hospital surveillance and implementation-related quantitative data will be collected using a structured questionnaire from various sources. Data for targeted practices shall be collected using observation checklists. An electronic data capture platform shall ensure timely and high-quality data collection and transmission. For qualitative data, semi-structured interview guides will be developed according to the domains, stakeholder roles, and responsibilities. The quantitative data collected at the sites shall be regularly uploaded to the cloud server with periodic backup. Data shall be checked regularly for completeness, consistency, range, and quality. The qualitative data will be transcribed and translated. The formative research components, stakeholders, data collection methods, and the corresponding sample sizes are shown in Table 2.

**Table 2:** Formative research components, stakeholders, data collection methods and sample size CRAb: carbapenem-resistant *Acinetobacter baumannii*; CSAb: carbapenem-sensitive *Acinetobacter baumannii*; GPS: global positioning system; HICC: hospital infection control committee; IPC: infection prevention and control; AMSP: antimicrobial stewardship Program; ICU: intensive care unit; BSI: bloodstream infection; SSI: surgical site infection; VAP: ventilator-associated pneumonia; CAUTI: catheter-associated urinary tract infection; CSSD: central sterilization and supply department

Study phase	Domain of enquiry	Settings/Stakeholders	Data collection methods	Sample size
Formative research phase	Acinetobacter baumannii and CRAb isolates and HAIs	Laboratory records/registers (for three months retrospective and three months prospective)	Record review (prospective and retrospective, combined)	All isolates
	Information about patients with CRAb & CSAb isolates	Clinical, investigations, treatment, referral pattern, sociodemography, GPS location (during three months)	Prospective, interview	All patients
	Assessment for IPC, HICC, and AMSP activities of hospitals	Hospital IPC, HICC, and AMSP practices, protocols, and functions over the last year	Document review and interaction	For each facility/hospital
	Patient flow tracking	Track the patients with HAI due to CRAb, CSAb, and no HAI (including BSI, SSI, VAP, CAUTI) (over three months prospectively)	Observation, record review, and interview	30 each
	Stakeholder perspectives	Healthcare providers (doctors, faculty, residents, and nurses) and support staff from different departments/wards/ICUs (medical and surgical) with high and low CRAb isolates	In-depth interviews (semi-structured guide)	Eight to 10 doctors, eight to 10 nurses, and four support staff
		Microbiologist		One to two
		Pharmacist		One to two
		Infection control nurse and officer		One to two
		HICC and AMSP committee members		Two to four
		CSSD in-charge		one
		Hospital administrator		One to two
Implementation phase	Acinetobacter baumannii and CRAb isolates and HAIs	Laboratory records/registers (continuous)	Active prospective surveillance	All isolates
	Information about patients with CRAb & CSAb isolates	Clinical investigations, treatment, referral pattern, sociodemography, and GPS location	Prospective, interview	All patients
	Periodic assessment for IPC, HICC, and AMSP activities of hospitals	Hospital IPC, HICC, and AMSP practices, protocols, and functions every six months	Document review and interaction	For each facility/hospital
	Patient flow tracking	Track the patients with HAI due to CRAb, CSAb, and no HAI (including BSI, SSI, VAP, CAUTI)	Observation, record review, and interview	30 each
	Intervention-specific activities	Hand hygiene practices, protocol adherence, and invasive procedure-linked and device handling practices (distributed over every quarter)	Observations	30 each
	Stakeholder perspectives	Healthcare providers (doctors, faculty, residents, and nurses) and support staff from the departments/wards/ICUs where interventions are implemented (every quarter and as needed)	In-depth interviews (semi-structured guide)	Four to six doctors, four to six nurses, and four staff
		Infection control nurse/officer		One to two
		HICC/AMSP committee		One to two
		Hospital administrator		One to two
Evaluation phase	Acinetobacter baumannii and CRAb isolates and HAIs	Laboratory records/registers (over 3 months)	Active prospective surveillance	All isolates
	Information about patients with CRAb & CSAb isolates	Clinical investigations, treatment, referral pattern, sociodemography, and GPS location (over three months)	Prospective, interview	All patients
	Periodic assessment for IPC, HICC, and AMSP activities of hospitals	Hospital IPC, HICC, and AMSP practices, protocols, and functions (during the last six months)	Document review and interaction	For each facility/hospital
	Patient flow tracking	Track the patients with HAI due to CRAb, CSAb, and no HAI (including BSI, SSI, VAP, CAUTI) (over three months)	Observation, record review, and interview	30 each
	Intervention-specific activities	Hand hygiene practices & protocol adherence, Invasive procedure-linked and device handling practices (over three months)	Observations	30 each
	Stakeholder perspectives	Healthcare providers (doctors, faculty, residents, and nurses) and support staff from different departments/wards/ICUs (medical and surgical) with high and low CRAb isolates	In-depth interviews (semi-structured guide)	Eight to 10 doctors, eight to 10 nurses, and four support staff
		Microbiologist		One to two
		Pharmacist		One to two
		Infection control nurse and officer		One to two
		HICC and AMSP committee members		Two to four
		CSSD in-charge		One
		Hospital administrator		One to two

Data analysis plan

Quantitative Analysis

Following quality check for duplication, redundancies, missing values, and outliers, the quantitative data shall be analyzed using descriptive statistics and expressed as proportions, means, and standard deviation (SD), or medians with interquartile range (IQR). The variables between the baseline, endline, and PDSA cycles/periods shall be compared using chi-square or paired t-tests, as appropriate. Bivariate analyses shall compare the CRAb and CSAb population characteristics to identify the risk factors and associations using Fisher's exact and Wilcoxon rank sum tests. Repeated measure Analysis of variance (ANOVA) shall be done for the dependent continuous variables with within-subject correlation. Linear mixed-effect models will perform the multivariate analysis for the same variables. Some variables' temporal pattern (if any) will be checked by autoregressive integrated moving average (ARIMA). We will perform a time-to-event analysis using the Cox proportional hazard model if any temporal pattern is indicated. The changes in indicators and parameters between baseline (pre-intervention) and endline (post intervention) and their statistical significance (p < 0.05) shall be derived. R software (version 4.5.1 or higher) [18] will be used for the data analysis and generation of plots.

Qualitative Analysis

Qualitative data shall be inductively analyzed using free listing, coding, axial coding, and cross-tabulation. Quantitative and qualitative data integration shall be done at the analysis level for each cycle and at the pooled level. Quantitative and qualitative data shall be reviewed to identify linkages and relationships for appropriate action at different levels.

Quantitative and qualitative data integration shall be done at the analysis level for each cycle and at the pooled level to identify linkages and/or relationships between different levels.

Genomic Analysis

The sequence reads shall undergo species identification and sequence typing using the multilocus sequence typing (MLST) Oxford scheme and comparison with the publicly available genome database to assign clonal complexes. The clonal groups shall be identified based on the SNP distances and Permanova variations. Pairwise SNP distance thresholds shall assist in identifying genetic relatedness, transmission tree generation, outbreak clustering, and transmission analysis (using GraphSNP, developed by Budi Permana at the Beatson Lab, University of Queensland, Australia, https://graphsnp.fordelab.com/)

Resistome analysis will be done to detect carbapenem resistance determinants for the isolates. Pairwise episode comparison and patient transfer network data shall assist in identifying the possible inter-facility transfer and/or importation cases.

Geospatial Analysis

**Figure 3:**
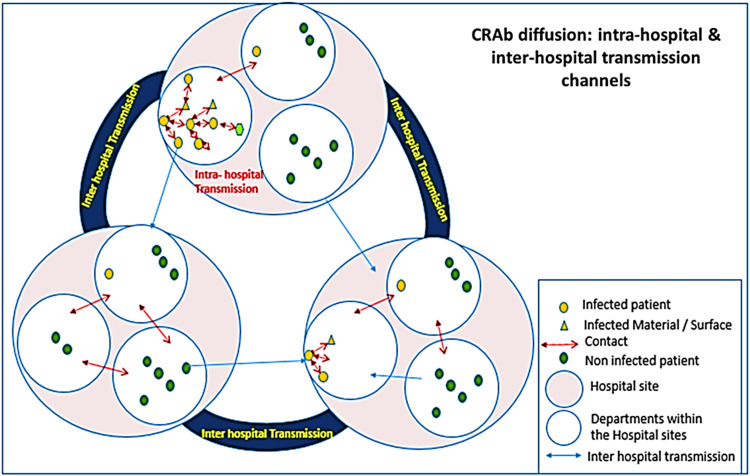
The geospatial epidemiological framework of CRAb transmission The figure has been created by Manoj K. Das The schematic diagram illustrates the different situational contexts for CRAb transmission, intra-facility (within the individual ward/unit/ICU and between the wards/units/ICUs) and inter-facility transmissions. CRAb: carbapenem-resistant *Acinetobacter baumannii*.

Multi-dimensional geospatial epidemiology techniques shall generate epidemiological patterns, including Bayesian kriging, kernel density maps, point pattern analysis, hub analysis, overlay analysis, and space-time cube analyses, to explore the spatiotemporal pattern of the variables of interest. The degree of similarity between spatial features will be calculated using spatial autocorrelation statistics, such as Moran's I or Geary's C, which will help identify clustering or dispersion patterns. The framework for mapping the intra- and inter-facility transmissions is shown in Figure 3.

Ethical considerations

This study will adhere to the ethical standards of the Good Clinical Practice Guidelines and the Indian Council of Medical Research's (ICMR) National Ethical Guidelines 2017 [19]. Ethical approvals have been received from all six participating institutes (approval numbers: KIIT/KIMS/IEC/1953/2025, T/EMF/Micro/2024-25/130, IEC/IMS.SH/SOA/2025/1018, 267/Chairman-IEC, 01/IEC, SLN MCH, and 77/10th IEC). Patient-related data will be collected following written informed consent and/or assent for minor patients. We will obtain the participants' consents for genomic sequencing and data-linkage risks. All individual interviews will be conducted, ensuring confidentiality. Anonymity and confidentiality of data shall be ensured while sharing and disseminating.

## Results

Since the study has not commenced, the results have yet to be obtained.

The primary outcome indicators shall entail (i) the number of CRAb incidences every quarter at all the study sites; (ii) the proportion of CRAb-associated HAIs in the wards/units/ICUs every quarter; and (iii) the proportion of CRAb-associated HAIs detected as potential inter-facility transmission. The secondary outcome indicators shall encompass (i) detection of HAIs (bloodstream infection (BSI), surgical site infection (SSI), ventilator-associated pneumonia (VAP), catheter-associated urinary tract infection (CAUTI)); (ii) rational antibiotic use; (iii) hand hygiene practices; (iv) invasive line and catheter insertion and handling practices; (v) unit-level sanitation (surface) and disinfection (reusable devices) practices; (vi) biomedical waste management; (vii) IPC, AMSP, and HICC surveillance; and (viii) knowledge and attitude status of care providers. Implementation outcome indicators shall include acceptability (aggregable for stakeholders), adoption (initiation by stakeholders), appropriateness (perceived fit and relevance for setting), feasibility (utilization in routine practice), penetration (used by stakeholders and units/wards/ICUs), and sustainability (maintenance of practices). Process outcome indicators will be used to monitor, optimize, and document the quality of model implementation. These will be specific to the strategies and interventions identified, and inputs for co-development and refinements.

## Discussion

The inter-facility patient transfers/referrals (within the state and regions) are frequent, which is associated with a high risk of HAIs and CRAb importation. These inter-facility patient transfers/referrals cannot be avoided. Thus, for effective prevention/control of HAI outbreaks and inter-facility transmission of CRAb, coordinated surveillance to identify the risk factors at the individual (patient risk-profiling), pathogen (phylogenetic lineage), and micro- and macro-system (processes/practices/behaviors/organization) levels across the network, and adopt evidence-based measures. As hospitals/facilities are part of the interconnected healthcare network, it is imperative that they are treated as part of the larger healthcare ecosystem and that synchronized actions/strategies are implemented for control/prevention of intra- and inter-facility HAI and CRAb transmissions. This proposed implementation research embedded with integrated laboratory-clinical-epidemiological-spatial-genomic surveillance within a state/regional hospital network will document the effect of contextualized interventions/solutions on CRAb-associated HAIs.

Strengths and limitations

This proposed implementation research, leveraging integrated laboratory-clinical-epidemiological-spatial-genomic surveillance approaches within an interconnected state-level hospital network, and the use of WGS data, are strengths of this study. The hospital-level, unit/ward/ICU-level, and individual-level inherent structural and procedural factors and activities with sizable financial implications may be amenable to change, which could affect the outcome. The pattern of inter-hospital transfers, case load, and CRAb isolates may vary beyond the project's limit. There is no baseline or background information, which could affect the power of the outcome, based on the sample size proposed.

## Conclusions

This is the first study on the phylogenetic and clonal analysis of HAIs due to CRAb in the state/region and for the intra- and inter-facility transmissions. The findings shall have implications for the HAI control and AMSP at the hospitals. Based on the findings, appropriate interventions and adaptations should be refined to reduce intra-facility transmissions, outbreaks, and importations of CRAb and other HAIs. The geospatial signatures of the horizontal transmission of CRAb will generate a new knowledge base for designing future containment and control measures for CRAb and other HAIs. We believe that the network of institutions will be leveraged to continue further research activities on India's antibiotic resistance.
